# Health care professionals’ attitudes towards population-based genetic testing and risk-stratification for ovarian cancer: a cross-sectional survey

**DOI:** 10.1186/s12905-017-0488-6

**Published:** 2017-12-16

**Authors:** Katie E. J. Hann, Lindsay Fraser, Lucy Side, Sue Gessler, Jo Waller, Saskia C. Sanderson, Madeleine Freeman, Ian Jacobs, Anne Lanceley, for the PROMISE study team

**Affiliations:** 1 Department of Women’s Cancer, EGA UCL Institute for Women’s Health, University College London, 74 Huntley Street, London, WC1E 6AU UK; 2 Department of Behavioural Science and Health, Institute of Epidemiology and Health Care, University College London, London, UK; 3 University Hospital Southampton NHS Foundation Trust, Southampton, UK; 4 Great Ormond Street Hospital, London, UK; 5 The University of New South Wales, Sydney, NSW Australia

**Keywords:** Health care professionals, Ovarian cancer, Genetic testing, Risk stratification

## Abstract

**Background:**

Ovarian cancer is usually diagnosed at a late stage when outcomes are poor. Personalised ovarian cancer risk prediction, based on genetic and epidemiological information and risk stratified management in adult women could improve outcomes. Examining health care professionals’ (HCP) attitudes to ovarian cancer risk stratified management, willingness to support women, self-efficacy (belief in one’s own ability to successfully complete a task), and knowledge about ovarian cancer will help identify training needs in anticipation of personalised ovarian cancer risk prediction being introduced.

**Methods:**

An anonymous survey was distributed online to HCPs via relevant professional organisations in the UK. Kruskal-Wallis tests and pairwise comparisons were used to compare knowledge and self-efficacy scores between different types of HCPs, and attitudes toward population-based genetic testing and risk stratified management were described. Content analysis was undertaken of free text responses concerning HCPs willingness to discuss risk management options with women.

**Results:**

One hundred forty-six eligible HCPs completed the survey: oncologists (31%); genetics clinicians (30%); general practitioners (22%); gynaecologists (10%); nurses (4%); and ‘others’. Scores for knowledge of ovarian cancer and genetics, and self-efficacy in conducting a cancer risk consultation were generally high but significantly lower for general practitioners compared to genetics clinicians, oncologists, and gynaecologists. Support for population-based genetic testing was not high (<50%). Attitudes towards ovarian cancer risk stratification were mixed, although the majority of participants indicated a willingness to discuss management options with patients.

**Conclusions:**

Larger samples are required to investigate attitudes to population-based genetic testing for ovarian cancer risk and to establish why some HCPs are hesitant to offer testing to all adult female patients. If ovarian cancer risk assessment using genetic testing and non-genetic information including epidemiological information is rolled out on a population basis, training will be needed for HCPs in primary care to enable them to provide appropriate support to women at each stage of the process.

**Electronic supplementary material:**

The online version of this article (10.1186/s12905-017-0488-6) contains supplementary material, which is available to authorized users.

## Background

Ovarian cancer is the sixth most common cancer among women in the UK [1]. Due to the lack of distinctive symptoms, most women are diagnosed at a late stage when mortality is high. Around 46% of women diagnosed with ovarian cancer in England and Wales survive 5 years or more; however, in the large proportion of women diagnosed with advanced stage disease, 5-year survival is only 10% [1]. Ovarian cancer screening within the general population is currently not available on the National Health Service (NHS) in the UK due to lack of definitive evidence that it decreases mortality through early detection [2].

The strongest genetic risk factors for ovarian cancer are mutations in the *BRCA1/2* genes. Clinical genetic testing for *BRCA1/2* gene mutations is currently available as a service on the NHS in the UK to individuals with a 10% or greater chance of carrying a *BRCA1/2* mutation [3] including those with high grade non-mucinous ovarian cancer, or those with a strong family history of breast and/or ovarian cancer. Once a pathogenic mutation is identified, testing can be offered to relatives to estimate their risk of developing the disease and to identify the most appropriate risk management strategy [4]. Approximately 10–15% of ovarian cancer cases are believed to be due to a *BRCA1/2* mutation [5, 6], however ~50% of individuals with a pathogenic *BRCA* mutation may not report a strong family history of cancer [7, 8]. These families may not be referred to clinical genetics even though current guidelines [9] recommend testing in these situations. Population based genetic testing could address this.

Progress in (1) scientific understanding of the genetic basis of different sub-types of ovarian cancer, as well as (2) the identification of common gene mutations that cumulatively may confer an increased risk, and (3) lower costs of genetic testing, mean that population-based risk stratification based on the combination of genetic and non-genetic information (e.g. family history, lifestyle) could become a viable option [10, 11]. The PROMISE research programme (Predicting Risk of Ovarian Malignancies, Improving Screening and Early detection, https://eveappeal.org.uk/our-research/our-research-programmes/promise-2016/) aims to improve early detection and risk management of ovarian cancer. PROMISE includes a feasibility study for a population-based risk stratified programme for ovarian cancer, utilising genetic testing. Risk-management strategies including information for those at low risk and screening or risk-reducing surgery for those at intermediate and high risk will then be offered accordingly. This population-based approach could improve early ovarian cancer detection by flagging up high-risk individuals for intensive screening, while reducing the number of unnecessary investigations carried out on low-risk individuals [12]. Recently published findings from the UK Familial Ovarian Cancer Screening Study (UKFOCSS) indicate that multimodal screening, involving 4-monthly blood tests for biomarker CA125 interpreted with the risk of ovarian cancer algorithm (ROCA), and secondary testing with transvaginal ultrasound, is highly sensitive and can detect ovarian cancer at an earlier stage [13]. Whilst risk-reducing surgery would still be the best option for women at high risk of ovarian cancer, screening could be offered to those who choose not to have surgery. The ability and willingness of health care professionals (HCPs) to support such a programme of population-based risk assessment and stratified management [11] needs to be established before it could be rolled out in the UK.

Evidence from Europe and the US suggests that HCPs in primary care generally have quite low levels of genetics knowledge [14, 15] and lack confidence in their ability to carry out tasks such as recording patients’ family histories of disease, discussing the risks and benefits of genetic testing and counselling patients about their test results [16–18]. It is unsurprising to find that oncologists and gynaecologists are more knowledgeable about genetics than general or family practitioners [19, 20]. It is encouraging to note that HCPs who completed training more recently appear to have more knowledge of genetics [21, 22], and greater confidence in their ability to explain genetic test results than those who had been practising for over 20 years [22]. HCPs’ knowledge and confidence in their ability to provide appropriate information is important, as it may influence referring behaviour [23]. However, there is little recent research investigating UK HCPs’ knowledge of cancer genetics or confidence in assessing cancer risk and discussing genetic testing with patients.

While HCPs acknowledge that genetic testing can help identify those at risk of potentially life-threatening diseases, personalise interventions, indicate when to offer screening and increase the ability to prevent certain cancers [15, 18, 24], several barriers have been identified [25, 26]. These include lack of time and clear referral and management guidelines [22, 24], as well as lack of clarity about what an abnormal genetic test result might mean for an individual patient’s cancer risk [25, 27]. Understandably, physicians have expressed apprehension about discussing genetic risk with patients if an effective treatment or intervention is not available [15, 28]. There is concern that adverse genetic test results might have a negative impact on patients’ psychological well-being [17, 22, 25]. Furthermore, both HCPs and patients have concerns about potential discrimination from insurers or employers following a genetic test result indicating increased disease risk [25, 27, 29].

This study aimed to investigate UK HCPs’ knowledge of ovarian cancer genetics and other risk factors, as well as self-efficacy (belief in one’s own ability to successfully complete a task [30]) in discussing cancer risk and genetic testing with patients, in order to identify professional training needs. Attitudes towards population-based genetic testing and stratified risk management strategies for ovarian cancer were also explored.

## Methods

An anonymous cross-sectional online survey was developed using the software Opinio as an effective method of reaching a convenience sample of HCPs [31]. This type of non-probability sampling involves drawing the sample from a readily available and convenient source of HCPs via groups and networks to which we had easy access. These included: British Gynaecological Cancer Society, National Forum of Gynaecological Oncology Nurses; the UK Cancer Genetics Group; Cancer, General Practice and Genetics Clinical Research Networks, The Royal College of General Practitioners; and the National Cancer Research Institute. We also recruited participants by ‘snowballing’ using a list of researcher contacts. Potential participants received an invitation from their professional organisation either by email with a link to the online survey, or by a similarly worded advertisement in a web-based professional news update. Some organisations sent a reminder email or advertisement with a link to the survey approximately 1 month after the initial invitation. Participants were eligible if they self-identified as a HCP based in the UK and the survey had relevance to their practice. HCPs who voluntarily accessed the survey, and completed and submitted it, did so in the knowledge that they were thereby giving consent for their anonymous responses to be included in the study. The participant information preface to the online survey explained these steps in the consent process and stated that since data was anonymous formal verbal or written consent was not required. The study was approved by UCL research ethics committee (project ID: 8053/002) and was open to participants for 3 months from March 2016.

The survey items were generated with reference to the literature on HCPs’ knowledge of and attitudes towards genetic testing and were refined by the multidisciplinary research team. A pilot was carried out in a sample of 6 HCPs and adjustments were made to the structure and order of questions based on this pilot. Participants were briefed about the PROMISE research programme to establish the context for this study, and questions were framed in relation to population-based genetic testing and risk stratification for women. (See Additional file 1 for the full survey and information provided to participants.)

Knowledge of ovarian cancer and genetics was measured using five True/False/Not sure questions and three multiple choice questions about the risk of ovarian cancer within (1) the general population and (2) those with a *BRCA1* or *BRCA2* mutation. Correctly answered questions were added to give a score of 0–8. The measure had adequate internal reliability (α = .691).

Seven items were included to assess HCPs’ self-efficacy in conducting a clinical cancer risk consultation. A four-point Likert-style response was used including the options “not at all confident”, “somewhat confident”, “quite confident” and “very confident”. Scores were produced by calculating the average of the 7 items to give a score between 1 and 4, where a higher score indicates higher levels of self-efficacy. The internal reliability was high for this measure (α = .936).

HCPs’ attitudes (perceived benefits and risks) towards hypothetical population-based genetic testing for ovarian cancer risk were measured with 7 items. Beliefs about risk stratification levels were explored with 3 items in which participants were asked to indicate the level of lifetime ovarian cancer risk (as a percentage) that you think is ‘low/intermediate/high risk’. Attitudes (perceived benefits and risks) towards risk stratification were measured with nine items after presenting the actual risk levels/thresholds set for the PROMISE feasibility study (low risk = 0 to 4.9% risk, intermediate risk = 5 to 9.9% risk, high risk = ≥10% risk). Three items were used to establish participants’ self-efficacy in communicating the stratified levels of risk. All attitude items used a 5-point Likert-style response scale to measure the extent to which participants agreed with each statement (strongly disagree to strongly agree).

HCPs’ willingness to tailor patient management according to risk was measured with 5 items, after presenting participants with the recommended management options from the PROMISE feasibility study. A 4-point Likert-style response was provided for participants to indicate their willingness (“yes, definitely” to “no, definitely not”). Participants were also presented with optional open-ended questions requesting further information on their reasons for their decisions.

Background and demographic information was collected on participants’ gender, age, ethnicity, current post, clinical setting, years in post and year of graduation. Further questions were used to elicit: whether they had learnt about genetic cancer risk during their training; how often they were involved in any aspect of assessing risk for ovarian and/or breast cancer; and whether they or any of their close relatives had been diagnosed with, or had had a risk assessment for, ovarian cancer or another cancer.

Frequencies were calculated for each item. Comparisons were made between completers’ and non-completers’ knowledge and self-efficacy scores using non-parametric Mann Whitney tests.

Participants were grouped by specialism: oncologists (including medical, clinical and gynaecological oncologists and oncology surgeons), cancer genetics clinicians (including clinical geneticists and genetic counsellors), General Practitioners (GPs) and gynaecologists. There were too few participants who self-identified as nurse specialists or ‘other’ to include in statistical comparisons. Differences between the groups of HCPs regarding relevant knowledge and self-efficacy to conduct a risk consultation were explored with Kruskal-Wallis tests and pairwise comparisons. Fisher’s exact tests compared HCPs’ willingness to offer genetic testing for ovarian cancer risk to all adult female patients after response categories were collapsed into three: agree/strongly agree, neutral, and disagree/strongly disagree. Bonferroni corrections were made for multiple testing. All statistical analyses were carried out using SPSS version 22.

Inductive content analysis [32] was performed on the free text responses to open-ended questions concerning participants’ willingness to discuss risk-management strategies with patients. Analysis was conducted by two researchers who independently read and made notes alongside the data (KH & MF). Themes were identified and a coding manual was produced. One researcher coded all data (KH), and the second researcher independently coded 10% of the data (MF). A Cohen’s κ calculation indicated that agreement in coding between the two researchers was substantial (according to Landis and Koch [33]), κ = .744 (95% CI = .689 to .862), *p* < .001. Ambiguous responses such as ‘as before’ or ‘as previous answer’ were not included in the analysis.

## Results

### Sample characteristics

In total, 253 potential participants opened the survey link and 149 (58.9%) completed the survey. Of the 149 participants who completed the survey, 3 were ineligible as it was unclear if they were registered HCPs, producing a final sample size of 146 participants. Table 1 shows the demographic characteristics of the participants. The majority of participants indicated they had learnt about inherited cancer risk during training, and over half were often involved in the referral or another part of the process of assessing risk for ovarian and/or breast cancer.

**Table 1:** Participant characteristics (*n* = 146)

	**N (%)**
Mean age (SD)	45.4 (8.8)
Gender	
Male	45 (30.8)
Female	99 (67.8)
Prefer not to say	2 (1.4)
Ethnicity	
White	122 (83.6)
Asian	11 (7.5)
Black	1 (0.7)
Mixed	2 (1.4)
Other	2 (1.4)
Prefer not to say	8 (5.5)
Current post	
General Practitioner	32 (21.9)
Genetics Specialist	44 (30.1)
Oncologist	45 (30.8)
Gynaecologist	15 (10.3)
Nurse specialist	6 (4.1)
Other	4 (2.7)
Years in post, Mean (SD)	11.7 (8.2)
Learnt about inherited cancer risk during training (yes)	108 (74.0)
Involvement in referral or any part of the process of assessing risk for ovarian and/or breast cancer risk	
Often	86 (58.9)
Sometimes	43 (29.5)
Rarely	15 (10.3)
Never	2 (1.4)
Personal or close family member with cancer diagnosis (yes)	52 (35.6)
Personal or close family had a cancer risk assessment (yes)	24 (16.4)

### Comparing survey completers and non-completers

It was not possible to calculate a response rate, as the number of potential participants that the survey invite reached was not known due to our non-probability sampling techniques including ‘snowballing’ where existing study participants recruit others by referring the study on within their professional network. However, we were able to compare key outcomes between HCPs who completed vs. did not complete the survey. Fifty-five individuals completed the self-efficacy questions and 32 completed the knowledge questions but did not complete the entire survey. The median score for perceived self-efficacy in conducting a cancer risk consultation was significantly higher among those who completed the survey (*Mdn* = 3.2, IR = 1.0) than those who did not (*Mdn* = 3.0, IR = 1.0), U = 4760.0, z = 2.035, *p* = .042, *r* = .14. Knowledge of ovarian cancer and genetics was also significantly higher among survey completers (*Mdn* = 7.0, IR = 3.00) than non-completers (*Mdn* = 5.0, IR = 3.75), U = 3361.5, z = 3.719, *p* < .001, *r* = 0.28.

### Self-efficacy in conducting a cancer risk consultation

Table 2 presents participants’ responses to each item measuring self-efficacy. The majority reported feeling ‘quite confident’ or ‘very confident’ in their abilities across all aspects of the consultation. The median score for the sample was 3.2 (IR = 1.0) out of 4, indicating quite high levels of self-efficacy.

**Table 2:** Self-efficacy in conducting a cancer risk consultation (*n* = 146)

	Not at all confident N (%)	Not very confident N (%)	Quite confident N (%)	Very confident N (%)
Initiate talking to patients about genetic testing for OC.	2 (1.4)	17 (11.6)	52 (35.6)	75 (51.4)
Record relevant information on a patient’s family history of cancer.	0	8 (5.5)	55 (37.7)	83 (56.8)
Respond to patients’ questions about OC risk based on family history.	4 (2.7)	30 (20.5)	60 (41.1)	52 (35.6)
Respond to patients’ questions about genetic testing for OC risk.	5 (3.4)	28 (19.2)	58 (39.7)	55 (37.7)
Explain lifetime cancer risk to patients.	5 (3.4)	36 (24.7)	50 (34.2)	55 (37.7)
Explain age-related cancer risk to patients.	4 (2.7)	32 (21.9)	69 (47.3)	41 (28.1)
Provide support to patients going through cancer risk assessment based on family history and genetic testing.	5 (3.4)	27 (18.5)	64 (43.8)	50 (34.2)

Significant differences in self-efficacy scores were found between oncologists, genetics specialists, GPs and gynaecologists, H(3) = 84.273, *p* < .001. GPs (*Mdn* = 2.4) had significantly lower scores than genetics specialists (*Mdn* = 3.9, p < .001, *r* = 1.05), oncologists (*Mdn* = 3.3, p < .001, *r* = 0.60) and gynaecologists (*Mdn* = 3.1, p < .001, *r* = 0.59). Genetics specialists had significantly higher self-efficacy scores than oncologists (*p* < .001, *r* = −0.46) and gynaecologists (*p* = .024, *r* = 0.37). No significant differences were found between gynaecologists’ and oncologists’ self-efficacy scores (*p* > .05). (See Supplementary File 2, Table S1.)

### Knowledge of ovarian cancer and genetics

Table 3 shows the number of correct responses to each of the knowledge questions for the whole sample and by HCP group. The median score for the whole sample was 7 (IR = 3.0) out of 8, indicating a high overall level of knowledge. Questions on the approximate risk of ovarian cancer in women with a *BRCA1* or *BRCA2* mutation were most frequently answered incorrectly.

**Table 3:** HCP ovarian cancer and genetics knowledge (*n* = 146)

	Total (*n* = 146)	GP (*n* = 32)	Genetics (*n* = 44)	Oncologists (*n* = 45)	Gynaecology (*n* = 15)	Nurse & other (*n* = 10)
	Correctly answered, N (%)					
A smear test is not designed to detect ovarian cancer (True)	143 (97.9)	32 (100.0)	43 (97.7)	44 (97.8)	15 (100)	9 (90.0)
Taking the contraceptive pill can increase a woman’s risk of developing ovarian cancer (False)	134 (91.8)	24 (75.0)	43 (97.7)	42 (93.3)	15 (100)	10 (100.0)
The majority of cases of ovarian cancer are caused by an inherited genetic mutation (False)	123 (84.2)	13 (40.6)	43 (97.7)	44 (97.8)	15 (100)	8 (80.0)
Paternal family history of cancer is as important as maternal family history of cancer when considering a patient’s risk of ovarian cancer (True)	108 (74.0)	10 (31.3)	42 (95.5)	37 (82.2)	10 (66.7)	9 (90.0)
A genetic test result that shows a patient has a variant of uncertain significance (VUS) indicates that the patient does not have an increased risk for ovarian cancer (False)	111 (76.0)	15 (46.9)	41 (93.2)	32 (71.1)	13 (86.7)	10 (100.0)
The average risk of a women developing ovarian cancer in her lifetime is approximately (2%)	115 (78.8)	13 (40.6)	44 (100)	37 (82.2)	14 (93.3)	7 (70.0)
The risk of a woman with a BRCA1 mutation developing ovarian cancer in her lifetime is approximately (30–60%)	102 (69.9)	13 (40.6)	40 (90.9)	32 (71.1)	11 (73.3)	6 (60.0)
The risk of a woman with a BRCA2 mutation developing ovarian cancer in her lifetime is approximately (10–30%)	91 (62.3)	8 (25.0)	36 (81.8)	29 (64.4)	10 (66.7)	8 (80.0)

When comparing the knowledge scores of GPs, genetics clinicians, gynaecologists and oncologists (*n* = 136), significant differences were found, H(3) = 73.233, *p* < .001. GPs (*Mdn* = 4.0) scored significantly lower than genetics clinicians (*Mdn* = 8.0, p < .001, *r* = 0.97), oncologists (*Mdn* = 7.0, p < .001, *r* = 0.61) and gynaecologists (*Mdn* = 7.0, p < .001, *r* = 0.69). It appears that while the majority of GPs correctly answered 2 non-genetic questions, far fewer correctly answered the genetics questions. Genetics clinicians also scored significantly higher than oncologists (*p* = .004, *r* = −0.36). No significant differences were found between gynaecologists and oncologists, or gynaecologists and genetics clinicians, although for the latter comparison this may be due to limited power (see Supplementary File 2, Table S1).

### Attitudes (perceived benefits and risks) towards hypothetical population-based genetic testing for ovarian cancer risk

Table 4 shows participants’ attitudes toward population-based genetic testing for ovarian cancer risk. Most HCPs agreed or strongly agreed that genetic testing would help identify those with a high risk of ovarian cancer. Participants were also in agreement that this would help patients make good healthcare decisions about managing their ovarian cancer risk. Less than 50% agreed that explaining genetic testing to patients would be too time consuming. However, the majority of participants believed that genetic testing could have a negative impact on some patients. In addition, responses were mixed with regard to whether participants were concerned that their patients would be discriminated against by insurers, and whether population-based genetic testing could be cost effective.

**Table 4:** Responses to items measuring attitudes towards population-based genetic testing for ovarian cancer risk (n = 146)

	Strongly disagree N (%)	Disagree N (%)	Neither N (%)	Agree N (%)	Strongly agree N (%)
It would help identify those with a high risk of ovarian cancer.	0	10 (6.8)	17 (11.6)	104 (71.2)	15 (10.3)
My patients could be discriminated against by insurers due to genetic testing results.	7 (4.8)	38 (26.0)	34 (23.3)	63 (43.2)	4 (2.7)
It could be cost effective in the long term.	1 (0.7)	27 (18.5)	50 (34.2)	57 (39.0)	11 (7.5)
Explaining genetic testing to patients would be too time consuming.	14 (9.6)	65 (44.5)	32 (21.9)	32 (21.9)	3 (2.1)
It would help patients make good healthcare decisions about managing risk.	1 (0.7)	13 (8.9)	23 (15.8)	99 (67.8)	10 (6.8)
It could have a negative impact on some of my patients.	1 (0.7)	16 (11.0)	21 (14.4)	94 (64.4)	14 (9.6)
*Willingness to offer population-based genetic testing*					
I would be willing to offer all my adult female patients genetic testing for OC risk.	8 (5.5)	33 (22.6)	35 (24.0)	53 (36.3)	17 (11.6)

### Willingness to offer population-based genetic testing

Just under half (47.9%) of the HCPs surveyed indicated that they would be willing to offer all their adult female patients genetic testing for ovarian cancer risk. A significant difference was found between oncologists, genetics clinicians, GPs and gynaecologists responses to this item, F(6) = 42.204, *p* < .001 (see Additional file 2 Table S2). Compared to genetics clinicians (18.2%), significantly more oncologists (68.9%) and GPs (50.0%) agreed with the statement ‘I would be willing to offer all my adult female patients genetic testing for ovarian cancer risk’. No significant difference was found between groups for the neutral response to this item. These results suggest that genetics clinicians were the least willing to offer genetic testing for ovarian cancer risk to all their adult female patients.

### Beliefs about ovarian cancer low, intermediate and high risk boundaries

Participants gave a variety of responses when asked to indicate what they thought the boundaries for ‘low risk’, ‘intermediate risk’ and ‘high risk’ for ovarian cancer are. The mode upper boundary for ‘low risk’ of ovarian cancer was 5% risk, however this response was only given by 37.7% of the sample and responses ranged from as low as 0% to 10%. The mode lower boundary for ‘high risk’ of ovarian cancer was 10% risk, which was the response of 39.0% of the sample, responses ranged widely from 5% up to 70%. The most frequently referred to boundaries for ‘intermediate risk’ was 5–10% risk, and responses ranged from 1 to 2% to 30–50% risk. The results indicate that the majority of participants’ perceptions of risk were not in-line with the clinically-informed boundaries adopted for the PROMISE feasibility study.

### Attitudes (perceived benefits and risks) towards risk stratification for ovarian cancer

Table 5 shows participants’ attitudes towards risk stratification for ovarian cancer. While the majority agreed that risk stratification for ovarian cancer would help identify those most in need of screening, approximately 80% also agreed that it would lead to ovarian cancer being missed in some patients.

**Table 5:** Responses to items measuring attitudes towards risk stratification (RS) for ovarian cancer (*n* = 146)

	Strongly disagree N (%)	Disagree N (%)	Neither N (%)	Agree N (%)	Strongly agree N (%)
RS would help identify those most in need of screening for ovarian cancer.	1 (0.7)	7 (4.8)	7 (4.8)	115 (78.8)	16 (11.0)
RS would lead to ovarian cancer being missed in some patients.	0	9 (6.2)	20 (13.7)	112 (76.7)	5 (3.4)
RS would give patients a sense of control over their health.	0	5 (3.4)	48 (32.9)	88 (60.3)	5 (3.4)
RS for ovarian cancer would make patients feel fatalistic about their health	2 (1.4)	57 (39.0)	77 (52.7)	10 (6.8)	0
Stratification into low risk would give a false sense of security.	2 (1.4)	38 (26.0)	40 (27.4)	65 (44.5)	1 (0.7)
Stratification into a low risk group would be reassuring.	0	13 (8.9)	28 (19.2)	104 (71.2)	1 (0.7)
Stratification into a group at high risk would have a negative impact on well being	1 (0.7)	31 (21.2)	51 (34.9)	59 (40.4)	4 (2.7)
Stratification into a group at intermediate risk would have a negative impact on well being	2 (1.4)	28 (19.2)	66 (45.2)	49 (33.6)	1 (0.7)
Stratification into a group at low risk would have a negative impact on well being	9 (6.2)	104 (71.2)	29 (19.9)	4 (2.7)	0
*Risk stratification consultation (communication) self-efficacy.*					
I am confident I could explain what ‘low risk’ means to patients in that group	0	7 (4.8)	10 (6.8)	97 (66.4)	32 (21.9)
I am confident I could explain what ‘intermediate risk’ means to patients in that group	0	9 (6.2)	15 (10.3)	93 (63.7)	29 (19.9)
I am confident I could explain what ‘high risk’ means to patients in that group	0	6 (4.1)	12 (8.2)	94 (64.4)	34 (23.3)

Participants had mixed views on how patients would react to being stratified by risk. Over 60% of the sample indicated that risk stratification would give patients a sense of control. Only 6.7% of participants believed risk stratification would cause patients to feel fatalistic about their health, although 53% were unsure. Most participants agreed that patients would feel reassured if stratified into a low risk group; fewer than half indicated that this would give patients a false sense of security. Fewer than half believed that being stratified into a high risk group or an intermediate risk group would have a negative impact on patients’ emotional well-being.

Most participants indicated that they would feel confident in explaining what ‘low risk’ (88.3% agreed/strongly agreed), ‘intermediate risk’ (83.6% agreed/strongly agreed) and ‘high risk’ means to patients (87.7% agreed/strongly agreed) (after they had been informed of the risk boundaries as set for the PROMISE feasibility study; see Supplementary File 1).

### Willingness to tailor patient management according to risk stratification based recommendations: Closed-ended questions

The majority of participants reported that they would be willing (probably or definitely) to discuss the suggested stratified interventions: 88.3% were willing to discuss information with those at low risk, 85.0% and 82.2% were willing to discuss screening (CA125 blood test every 4 months and an annual ultrasound scan) with patients at intermediate and high risk respectively, and 84.3% and 90.4% were willing to discuss surgery with intermediate and high risk patients respectively (see Additional file file 2: Table S3).

### Reasons for being willing/not willing to tailor patient management according to risk stratification-based recommendations: Open-ended questions

The optional free-text responses giving reasons for willingness to discuss the various interventions were approximately one or two sentences in length and were provided initially by 121 participants. Frequently reported reasons for being unwilling to discuss information with low risk patients were practical, such as lack of time and resources. Reticence about discussing screening with patients was mostly because it has not yet been shown to be effective, while reluctance to discuss surgery was due to some HCPs not considering this to be part of their job.

The most frequently given reasons for willingness to discuss symptom awareness and providing lifestyle advice was that this capitalised on a health promotion opportunity and would be useful for patients. Willingness to discuss screening was often described as being part of their job and they already offered similar tests to patients. Willingness to discuss surgery with patients was underpinned by the belief that it would benefit patients by reducing risk and preventing ovarian cancer. Several participants gave ‘conditional’ responses, for example they would discuss screening with patients if evidence proved its effectiveness or they would discuss surgery depending on patient characteristics such as age. See Additional file file 2: Tables S4 and S5 for a full list of the themes identified from participants’ responses, supporting quotes, and the number of times these themes appeared.

## Discussion

This study examined UK HCPs’ knowledge of ovarian cancer and genetics, their self-efficacy in conducting a cancer risk consultation, and their attitudes towards a programme using population-based genetic testing and risk stratified management for ovarian cancer. Survey responses reflected what the practitioners anticipated they would do in specific situations, not their actual practices. Overall, participants’ knowledge of ovarian cancer and related genetics was high and this was reflected in clinicians’ belief that they could effectively conduct a cancer risk consultation. However, substantial differences in knowledge were identified between the various specialists, with lower scores for GPs. This pattern of results is consistent with previous research in Europe and the US indicating lower knowledge of genetics among primary HCPs compared to more specialised HCPs [15, 19, 20]. The GPs’ results are similar to an earlier report [14] that 56.9% of primary care physicians knew that the majority of ovarian cancer cases are sporadic and 34.5% knew that maternal history was not more important than paternal history when considering cancers that mostly affect women, compared to 40.6and 31.3% of GPs in the current study. Significant differences between HCP disciplines were also identified regarding self-efficacy to conduct a cancer risk consultation, and GPs were again found to have lower scores. Despite this, the results indicate that the majority of participants were at least “quite confident” in their ability to record relevant information on patients’ family history of cancer, an improvement on findings from earlier research on recording family histories of disease [17, 28].

These results are perhaps unsurprising since GPs deal with a wide variety of health conditions, whilst genetics clinicians, oncologists, and gynaecologists have specialist training in genetics and/or cancer. Nonetheless, the findings highlight the need to improve GPs’ knowledge of ovarian cancer genetics and self-efficacy in discussing genetics and risk with patients for the successful introduction of population-based genetic testing for ovarian cancer risk in the UK. If genetic testing were offered clinically on a population basis it would be important for those found to be carrying a BRCA gene mutation or with a strong family history of cancer to receive counselling by genetics specialists. The service delivery model will need to be worked out, but primary care providers may well be responsible for ensuring patients from the general population (without a cancer diagnosis) make an informed decision about undergoing genetic testing, and in the ensuing risk assessment and stratification processes. HCPs including GPs will need to have a broad understanding of genetics and cancer, be able to record patient family history and lifestyle factors, use risk tools, interpret risk and communicate risk in a jargon-free manner. Furthermore, they will need to be aware of and able to manage patient anxieties and offer appropriate risk management options [34].

In parallel with improving the skills and knowledge of non-genetics clinicians and due to time pressures, tools such as decision aids could be useful for helping patients reach informed decisions about whether to have a cancer risk assessment and their risk management options. Decision aids (e.g. leaflets, websites) can increase patient knowledge about treatment options and can be more effective than usual care (such as having an appointment with a HCP alone) [35, 36]. Decision aids, which provide information and help patients to consider their personal values in relation to a health decision, can also help manage expectations and reduce decisional conflict [35].

Attitudes towards population-based genetic testing for ovarian cancer risk were mixed in the present study. While the majority of HCPs acknowledged the potential benefits for patients, several felt that genetic testing could negatively impact some patients and nearly half believed patients could be discriminated against by insurers based on test results; these findings are in line with previous research, although to a lesser extent [27, 37]. Since 2001, the Concordat and Moratorium (in place until November 2019) [38] has protected the interests of insurers and patients in the UK. These agreements, put together by the Government and the Association of British Insurers, stipulate that patients who have undergone predictive genetic testing do not have to reveal test results to insurers unless applying for life insurance over £500,000 or testing for Huntington’s disease. It is possible that HCPs are not aware of this agreement or that they anticipate that in the future it will change and potentially no longer provide the same protection to patients.

Importantly, fewer than half of the HCPs surveyed agreed that they ‘would be willing to offer all my adult patients genetic testing for ovarian cancer risk’. Of particular note is that, only 18% of genetics specialists agreed with this statement. Their reservations about testing may be because their professional knowledge makes them acutely aware that genetic tests may generate variants of unknown significance (VUS). A VUS is a change in the gene sequence where any association with an increased cancer risk is unclear or unknown. It is estimated that around 1 in 20 of *BRCA1* and *BRCA2* full screens in UK regional genetics laboratories will result in a VUS being identified [39]. Given that the chance of identifying a pathogenic mutation in full *BRCA1* and *BRCA 2* screening in the UK population is around 1 in 400, it is more likely that a VUS will be found if population testing is adopted [40]. Whilst international efforts to better characterise VUSs will likely result in the VUS rate decreasing in *BRCA1* and *BRCA2*, this is likely to take longer for other, less well characterised, genes such as *RAD51 C* and *D* and *BRIP1*. Here the VUS rate may remain significant, making advice to families difficult. The other concern for geneticists is that mutations identified on a population rather than family history basis, may have lower penetrance. Again, this raises difficulties in quantifying ovarian cancer risk and for subsequent management advice. For example, the *BRCA1 R1699Q* variant [41] appears to be a low-moderate penetrance *BRCA1* mutation, necessitating different risk management advice. Geneticists may also be more cautious because of the possibility of unintentionally identifying gene changes that increase the risk of diseases other than ovarian cancer. These incidental findings can arise as a result of the technologies used in population based panel testing. Furthermore, compared to many other HCPs, geneticists care for families rather than individuals and are cognizant of the implications of genetic test results for family members, as well as for the patient. Traditionally, genetic counselling has been attuned to the psychosocial as well as medical aspects of a patient’s care, and there is an awareness of the potential adverse psychological outcomes in patients who have a “bad news” predictive genetic test result [42, 43].

Increasingly, genetic testing is offered within gynae-oncology services to ovarian cancer patients with high-grade non-mucinous ovarian cancer, irrespective of their family history of cancer. Evidence suggests that this ‘mainstream’ approach is effective at identifying gene carriers who would not otherwise have been tested [44]. The willingness amongst oncologists to offer population-based genetic testing to all adult female patients may reflect the move towards mainstreaming *BRCA1/2* genetic testing within ovarian cancer care. Further work is needed to clarify the factors influencing UK HCPs’ willingness to offer population-based genetic testing.

Attitudes towards risk stratification for ovarian cancer were also mixed, though generally positive. Most participants agreed that risk stratification would help identify those most in need of screening and would give patients a sense of control over their health. They disagreed that risk stratification would make patients feel fatalistic about their health and over half did not agree that stratification into a low risk group would give patients a false sense of security, which accords with the views of women in previous studies [45]. Our finding that fewer than half of the HCPs believed that stratification into a high or intermediate risk group would have a negative impact on patients’ well-being may reflect awareness that patients who undergo genetic testing for cancer risk do not tend to experience adverse psychological consequences in the long-term [46].

Whilst most participants’ perceptions of low, intermediate and high risk boundaries were found to initially not be in line with those set by the PROMISE feasibility study, after revealing these boundaries the majority of HCPs surveyed reported that they would be confident in explaining what low, intermediate and high risk meant to patients in those groups. Although other research has found that oncologists and surgeons feel confident in reporting risk to patients [47], Nippert et al. [4] report in their European survey that most UK based GPs felt genetic specialists should explain genetic test results to patients. The communication of a health risk to patients with low health literacy or poor numerical skills can be particularly difficult [48].

The majority of participants indicated that, if population based risk assessment and stratification were offered, they would be willing to discuss symptom awareness and lifestyle advice with low risk patients, and screening and surgery with intermediate and high risk patients. Some identified a need for training and guidance. Willingness to discuss screening tests (CA125 blood tests and scans of the ovaries) with intermediate and high risk patients may reflect familiarity with their use as diagnostic tests for ovarian cancer. Some participants indicated they would not be willing to discuss ovarian cancer screening because it has not been shown to save lives, whereas others indicated willingness to discuss it on the condition that it is proven to be effective. These findings are unsurprising considering the publication of mortality results from the UK Collaborative Trial of Ovarian Cancer Screening (UKCTOCS) just a few months prior to the survey going live. Results from the randomised-controlled trial indicated that ovarian cancer screening using CA125 (interpreted with the ROCA) and ultrasound scans in a general population sample of post-menopausal women show high sensitivity, specificity, acceptability, and compliance, but at initial reporting the mortality impact did not reach statistical significance [2]. Recent results from the second phase of the UKFOCSS [13] were not available at the time of the survey and therefore will not have influenced the views of our survey participants. This trial found that ROCA-based screening for ovarian cancer of women at high risk (≥10% risk) was highly sensitive and resulted in a stage shift, indicating that it could be provided as an option to women who are not yet ready to undergo prophylactic surgery. However, the mortality impact could not be assessed as this was not a randomised trial with a control arm.

Recent studies suggest that risk-reducing bilateral salpingo-oophorectomy (RRSO) would be cost effective in postmenopausal women at ≥5% risk and premenopausal women over 40 years of age at ≥4% risk [49, 50]. Current UK practice is that women with a lifetime risk for ovarian cancer ≥10%, who have completed childbearing can be offered RRSO between age 35–40 years [51]. Interestingly, the majority of HCPs in the current study indicated that they would be willing to discuss RRSO with women at ‘intermediate risk’ of ovarian cancer (5–9.9% risk), although some explained that this discussion would be dependent on characteristics of the patient (e.g. age) or medical guidelines. Current guidelines [52, 53] indicate that RRSO may be considered for women who carry mutations in intermediate risk genes e.g. RAD51 and BRIP1 between age 45–50 or earlier. Only two participants indicated they would not be willing to discuss surgery with women at ‘intermediate risk’ as they felt RRSO was only appropriate for high risk women. However, the results reflect what HCPs anticipate they would do in a hypothetical scenario, rather than their actual actions or current practice. Further work is needed to tease out HCPs views of RRSO in women with a lower than 10% risk of ovarian cancer, as willingness to discuss RRSO with ‘intermediate risk’ patients may reflect a lack of knowledge of the current conditions under which RRSO is offered. This is particularly pertinent for genes such as RAD51 and BRIP1, where the risk may be 5–10%.

The small sample size of this survey study restricted the inter group comparisons and the ability to control for confounding factors. Comparisons could not be made on the demographics of those who completed and did not complete the survey (those who did not complete the survey did not answer the demographic questions). Due to the small sample size and because participants were active members of professional organisations, our findings may not extend to the wider HCP community. Our participants are likely to have better knowledge of, interest in, and professional experience with ovarian cancer and genetics. Furthermore, as the study was conducted with HCPs in the UK the results cannot be generalised to HCPs working in other countries where healthcare systems and genetic services are different. Finally, due to the methods of recruitment used it was not possible to calculate a response rate for this study. Despite these limitations, significant differences were detected between the different HCP groups, and fewer than half the participants stated that they would be willing to offer all female patients genetic testing for ovarian cancer risk, thus providing potentially important insights and testable hypotheses for future research.

## Conclusion

To our knowledge, this is the first UK survey of HCPs’ attitudes towards a programme using population-based genetic testing and stratified interventions for ovarian cancer. It provides novel insights into the acceptability of such a programme. If risk assessment and stratified management are to be used more widely to help improve early detection of ovarian cancer, more research is needed to identify potential barriers to their provision by HCPs and training needs.

## Additional files

Additional file 1: Survey Questions (DOCX 44 kb)

Additional file 2: Table S1. Comparisons between healthcare specialists’ ovarian cancer and genetics knowledge and self-efficacy to conduct a cancer risk consultation. **Table S2.** Comparisons between healthcare specialists willingness to offer all adult female patients genetic testing for ovarian cancer risk. **Table S3.** Willingness to discuss risk stratified interventions with patients. **Table S4.** Reasons for lack of willingness to discuss suggested interventions. **Table S5.** Reasons for lack of willingness to discuss suggested interventions (DOCX 31 kb)

## Abbreviations

GP: General Practitioner
HCP: Health care professional
NHS: National Health Service
PROMISE: Predicting Risk of Ovarian Malignancies, Improving Screening and Early detection
ROCA: Risk of Ovarian Cancer Algorithm
UKCTOCS: UK Collaborative Trial of Ovarian Cancer Screening
UKFOCSS: UK Familial Ovarian Cancer Screening Study

## References

[1] Ovarian Cancer Statistics [http://www.cancerresearchuk.org/health-professional/cancer-statistics/statistics-by-cancer-type/ovarian-cancer#heading-Zero]. Accessed October 2017.

[2] Jacobs IJ, Menon U, Ryan A, Gentry-Maharaj A, Burnell M, Kalsi JK (2016). Ovarian cancer screening and mortality in the UK collaborative trial of ovarian cancer screening (UKCTOCS): a randomised controlled trial. Lancet.

[3] Slade I, Riddell D, Turnbull C, Hanson H, Rahman N (2015). Development of cancer genetic services in the UK: a national consultation. Genome Med.

[4] Nippert I, Julian-Reynier C, Harris H, Evans G, van Asperen CJ, Tibben A (2014). Cancer risk communication, predictive testing and management in France, Germany, the Netherlands and the UK: general practitioners’ and breast surgeons’ current practice and preferred practice responsibilities. J Community Genet..

[5] Zhang S, Royer R, Li S, McLaughlin JR, Rosen B, Risch HA (2011). Frequencies of BRCA1 and BRCA2 mutations among 1,342 unselected patients with invasive ovarian cancer. Gynecol Oncol.

[6] Pal T, Permuth-Wey J, Betts JA, Krischer JP, Fiorica J, Arango H (2005). BRCA1 and BRCA2 mutations account for a large proportion of ovarian carcinoma cases. Cancer.

[7] Manchanda R, Loggenberg K, Sanderson S, Burnell M, Wardle J, Gessler S, et al. Population testing for cancer predisposing BRCA1/BRCA2 mutations in the Ashkenazi-Jewish community: A randomized controlled trial. J. Natl. Cancer Inst. 2015; 107(1):dju379. 10.1093/jnci/dju379 PMC4301703 25435541

[8] Gabai-Kaparaa E, Lahadb A, Kaufmand B, Friedmane E, Segevg S, Renbauma P (2014). Population-based screening for breast and ovarian cancer risk due to BRCA1 and BRCA2. Proc Natl Acad Sci.

[9] National Institute for Clinical Excellence (NICE) (2013). Familial breast cancer: classification, care and managing breast cancer and related risks in people with a family history of breast cancer..

[10] Dent T, Jbilou J, Rafi I, Segnan N, Törnberg S, Chowdhury S (2013). Stratified cancer screening: the practicalities of implementation. Public Health Genomics.

[11] Pashayan N, Pharoah P (2012). Population-based screening in the era of genomics. Pers. Med..

[12] Hall AE, Chowdhury S, Hallowell N, Pashayan N, Dent T, Pharoah P (2013). Implementing risk-stratified screening for common cancers: a review of potential ethical, legal and social issues. J Public Health.

[13] Rosenthal AN, Fraser LSM, Philpott S, Manchanda R, Burnell M, Badman P, et al. Evidence of stage shift in women diagnosed with ovarian cancer during phase II of the United Kingdom Familial Ovarian Cancer Screening Study Journal of Clinical Oncology. 2017;1-35(13):1411–20. 10.1200/JCO.2016.69.9330 PMC5455461 28240969

[14] Cohn J, Blazey W, Tegay D, Harper B, Koehler S, Laurent B (2014). Physician risk assessment knowledge regarding BRCA genetics testing. J Cancer Educ.

[15] Marzuillo C, De Vito C, Boccia S, D’Addario M, D’Andrea E, Santini P (2013). Knowledge, attitudes and behavior of physicians regarding predictive genetic tests for breast and colorectal cancer. Prev Med.

[16] Nippert I, Harris HJ, Julian-Reynier C, Kristoffersson U, Leo P, Anionwu E (2011). Confidence of primary care physicians in their ability to carry out basic medical genetic tasks—a European survey in five countries—part 1. J Community Genet..

[17] Suchard MA, Yudkin P, Sinsheimer JA, Fowler GH (1999). General practitioners’ views on genetic screening for common diseases. Br J Gen Pract.

[18] Birmingham WC, Agarwal N, Kohlmann W, Aspinwall LG, Wang M, Bishoff J (2013). Patient and provider attitudes toward genomic testing for prostate cancer susceptibility: a mixed method study. BMC Health Serv Res.

[19] Escher M, Sappino AP (2000). Primary care physicians’ knowledge and attitudes towards genetic testing for breast-ovarian cancer predisposition. Ann Oncol.

[20] Wideroff L, Vadaparampil ST, Greene MH, Taplin S, Olson L, Freedman AN (2005). Hereditary breast/ovarian and colorectal cancer genetics knowledge in a national sample of US physicians. J Med Genet.

[21] Baars MJ, Henneman L, ten Kate LP (2005). Deficiency of knowledge of genetics and genetic tests among general practitioners, gynecologists, and pediatricians: a global problem. Genet. Med..

[22] Acton RT, Burst NM, Casebeer L, Ferguson SM, Greene P, Laird BL (2000). Knowledge, attitudes, and behaviors of Alabama’s primary care physicians regarding cancer genetics. Acad Med.

[23] Gray SW, Hicks-Courant K, Cronin a, Rollins BJ, Weeks JC. Physicians’ attitudes about multiplex tumor genomic testing. J Clin Oncol. 2014;1, 32(13):1317–23. 10.1200/JCO.2013.52.4298 PMC3992721 24663044

[24] Bonter K, Desjardins C, Currier N, Pun J, Ashbury FD. Personalised medicine in Canada: a survey of adoption and practice in oncology, cardiology and family medicine. BMJ open. 2011; 1(1):e000110. doi:10.1136/bmjopen-2011-000110. 10.1136/bmjopen-2011-000110 PMC3191410 22021765

[25] Falahee M, Simons G, Raza K, Stack RJ. Healthcare professionals’ perceptions of risk in the context of genetic testing for the prediction of chronic disease: a qualitative metasynthesis. Journal of Risk Research 2016:1-38.

[26] Suther S, Goodson P (2003). Barriers to the provision of genetic services by primary care physicians: a systematic review of the literature. Genet Med.

[27] Freedman AN, Wideroff L, Olson L, Davis W, Klabunde C, Srinath KP (2003). US physicians’ attitudes toward genetic testing for cancer susceptibility. Am J Med Genet.

[28] Watson EK, Shickle D, Qureshi N, Emery J, Austoker J (1999). The ‘new genetics’ and primary care: GPs’ views on their role and their educational needs. Fam Pract.

[29] Chowdhury S, Dent T, Pashayan N, Hall A, Lyratzopoulos G, Hallowell N (2013). Incorporating genomics into breast and prostate cancer screening: assessing the implications. Genet Med.

[30] Bandura A (1977). Self-efficacy: toward a unifying theory of behavioural change. Psychol Rev.

[31] Braithwaite D, Emery J, de Lusignana S, Sutton S (2003). Using the internet to conduct surveys of health professionals: a valid alternative?. Fam Pract.

[32] Elo S, Kyngas H (2008). The qualitative content analysis process. J Adv Nurs.

[33] Landis JR, Koch GG (1977). The measurement of observer agreement for categorical data. Biometrics.

[34] Chowdhury S, Henneman L, Dent T, Hall A, Burton A, Pharoah P (2015). Do health professionals need additional competencies for stratified cancer prevention based on genetic risk profiling?. J Personalised Med.

[35] Stacey D, Legare F, Lewis K, Barry MJ, Bennett CL, Eden KB et al. Decision aids for people facing health treatment or screening decisions. Cochrane Data Base of Systematic Reviews. 2017;4:CD001431. DOI:10.1002/14651858.CD001431.pub5. 10.1002/14651858.CD001431.pub5 PMC6478132 28402085

[36] O’Brien MA, Whelan TJ, Villasis-Keever M, Gafni A, Charles C, Roberts R, et al. Are cancer-related decision aids effective? A systematic review and meta-analysis. 27. 2009;20-27(6):974–85. 10.1200/JCO.2007.16.0101 19124808

[37] Pichert G, Dietrich D, Moosmann P, Zwahlen M, Stahel RA, Sappino AP (2003). Swiss primary care physicians’ knowledge, attitudes and perception towards genetic testing for hereditary breast cancer. Familial Cancer.

[38] Department of Health: Concordat and Moratorium on Genetics and Insurance: 2014. In Edited by Health Do; 2014.

[39] Eccles DM, Mitchell G, Monteiro ANA, Schmutzler R, Couch FJ, Spurdle AB (2015). BRCA1 and BRCA2 genetic testing – pitfalls and recommendations for managing variants of uncertain clinical significance. Ann Oncol.

[40] Maxwell KN, Domchek SM, Nathanson KL, Robson ME (2016). Population frequency of germline BRCA1/2 mutations. J Clin Oncol.

[41] Spurdle AB, Whiley PJ, Thompson B, Feng B, Healey S, Brown MA (2012). BRCA1 R1699Q variant displaying ambiguous functional abrogation confers intermediate breast and ovarian cancer risk. J Med Genet.

[42] Lumish HS, Steinfeld H, Koval C, Russo D, Levinson E, Wynn J, et al. Impact of Panel Gene Testing for Hereditary Breast and Ovarian Cancer on Patients. J. Genet. Couns.. 2017; [Epub ahead of print]. 10.1007/s10897-017-0090-y PMC7250529 28357778

[43] Lapointe J, Dorval M, Noguès C, Fabre R, GENEPSO Cohort, Julian-Reynier C. Is the psychological impact of genetic testing moderated by support and sharing of test results to family and friends? Familial Cancer 2013; 12(4):601-610. 10.1007/s10689-013-9621-3 23475556

[44] George A, Riddell D, Seal S, Talukdar S, Mahamdallie S, Ruark E, et al. Implementing rapid, robust, cost-effective, patient-centred, routine genetic testing in ovarian cancer patients. Sci Rep. 2016;6 10.1038/srep29506 PMC4942815 27406733

[45] Meisel SF, Side L, Fraser L, Gessler S, Wardle J, Lanceley A (2013). Population-based, risk-stratified genetic testing for ovarian cancer risk: a focus group study. Health Genomics.

[46] Heshka JT, Palleschi C, Howley H, Wilson B, Wells PS (2008). A systematic review of perceived risks, psychological and behavioral impacts of genetic testing. Genet Med.

[47] Janz NK, Leinberger RL, Zikmund-Fisher BJ, Hawley ST, Griffith K, Jagsi R (2015). Provider perspectives on presenting risk information and managing worry about recurrence among breast cancer survivors. Psycho-Oncology.

[48] Apter AJ, Paasche-Orlow MK, Remillard JT, Bennett IM, Ben-Joseph EP, Batista RM (2008). Numeracy and communication with patients: they are counting on us. J Gen Intern Med.

[49] Manchanda R, Legood R, Antoniou AC, Gordeev VS, Menon U (2016). Specifying the ovarian cancer risk threshold of ‘premenopausal risk-reducing salpingooophorectomy’ for ovarian cancer prevention: a cost-effectiveness analysis. J Med Genet.

[50] Manchanda R, Legood R, Pearce L, Menon U (2015). Defining the risk threshold for risk reducing salpingo-oophorectomy for ovarian cancer prevention in low risk postmenopausal women. Gynecol Oncol.

[51] Hartmann LC, Lindor NM (2016). The role of risk-reducing surgery in hereditary breast and ovarian cancer. N Engl J Med.

[52] Daly MB, Pilarski R, Berry M, Buys SS, Farmer M, Friedman S (2017). NCNN guidelines insights: genetic/familial high-risk assessment: breast and ovarian, version 2. 2017. J Natl Compr Cancer Netw.

[53] Paluch-Shimon S, Cardoso F, Sessa C, Balmana J, Cardoso MJ, Gilbert F (2016). Prevention and screening in BRCA mutation carriers and other breast/ovarian hereditary cancer syndromes: ESMO clinical practice guidelines for cancer prevention and screening. Ann Oncol.

