# Supplementary material for: Health care professionals’ attitudes towards population-based genetic testing and risk-stratification for ovarian cancer: a cross-sectional survey
Source: BMC Womens Health. 2017 Dec 16;17:132. doi: 10.1186/s12905-017-0488-6 (PMC5732525; doi:10.1186/s12905-017-0488-6)
Supplement: Supplementary file 1 — Survey Questions (DOCX 44 kb) [file 12905_2017_488_MOESM1_ESM.docx]

**Background information**
PROMISE (Predicting Risk of Ovarian Malignancies, Improved Screening and Early detection) is an international collaborative programme being carried out at University College London, Cambridge University, the University of Southern California and Harvard Medical School under the direction of Professor Ian Jacobs (Chief Investigator) at the University of Manchester. The study is funded by the Eve Appeal and Cancer Research UK.

PROMISE aims to develop an ovarian cancer risk prediction programme offering stratified screening and intervention according to lifetime level of risk. The programme will involve population-based genetic testing* and risk prediction based on a variety of information about the individual. Women will then be stratified** into one of three groups representing their current estimated lifetime risk of developing ovarian cancer - low risk, intermediate risk, high risk – and managed accordingly.

**What is the purpose of this survey?**
The survey aims to find out about healthcare professionals' confidence in discussing ovarian cancer and genetics with patients, knowledge of ovarian cancer and related genetics, and views of the proposed risk prediction and stratified screening/interventions for ovarian cancer. This study is important as it will help us to understand healthcare professionals' acceptance and attitudes towards programmes of risk prediction and stratification for cancer.

The survey should take around 15 minutes.

**What will happen with my data?**
The data you provide to us will be anonymous as we will not be asking for identifying information, such as your name or address. The data will be kept confidential and stored, processed and destroyed in line with the Data Protection Act (1998). We may publish our findings from this survey.

**Important**
Participation in this survey is completely voluntary and you are free to withdraw at any time by simply exiting the survey. However, once you have submitted your answers we may not be able to withdraw your data as it will not be identifiable. Please note that once you have moved on from a section you will not be able to go back to review your answers, please do not use the 'back' button on your browser.

**By completing the survey you are indicating that you have read and understood the above information and you are providing consent for your participation.**

If you would like further information please do not hesitate to contact us:
Research assistant – Katie Hann, k.hann@ucl.ac.uk


*Population-based genetic testing would, in this circumstance, involve offering all adult females (18+) genetic testing for ovarian cancer related genetic mutations and variants that increase ovarian cancer risk, regardless of family history of cancer.

**Risk stratification involves identifying a person's chances of developing a disease (in this case ovarian cancer) and allocating them to a risk group on the basis of this information.

**OVARIAN CANCER AND GENETICS: CONFIDENCE**

**To start off with, we'd like to know how confident you feel talking to your patients about ovarian cancer and genetics. Some clinicians will already be doing this on a regular basis, others won't and might feel less confident about doing it. We're interested in your views.**

1. How confident do you feel in your ability to:

|  | Not at all confident | Not very confident | Quite confident | Very confident |
| --- | --- | --- | --- | --- |
| Initiate talking to patients about ovarian cancer? |  |  |  |  |
| Record relevant information on a patient’s family history of cancer? |  |  |  |  |
| Respond to patients’ questions about ovarian cancer risk based on family history? |  |  |  |  |
| Respond to patients’ questions about genetic testing for ovarian cancer risk? |  |  |  |  |
| Explain lifetime cancer risk to patients? |  |  |  |  |
| Explain age-related cancer risk to patients? |  |  |  |  |
| Provide support to patients going through cancer risk assessment based on their family history of cancer and genetic testing? |  |  |  |  |

**OVARIAN CANCER AND GENETICS**

Now we’d like to know what you think about ovarian cancer screening and early detection.

2. Please indicate the extent to which you disagree or agree with the following general statements about ovarian cancer.

|  | Strongly disagree | Disagree | Neither disagree or agree | Agree | Strongly agree |
| --- | --- | --- | --- | --- | --- |
| More should be done to detect ovarian cancer in women earlier. |  |  |  |  |  |
| There are reliable methods of screening for ovarian cancer. |  |  |  |  |  |
| Genetic test results are unreliable for predicting cancer risk. |  |  |  |  |  |
| Not much can be done to help detect ovarian cancer earlier. |  |  |  |  |  |
| There are effective treatments available to help prevent ovarian cancer. |  |  |  |  |  |

**POPULATION-BASED GENETIC TESTING**

We’d like you to think about population-based genetic testing for ovarian cancer risk. Imagine that this was introduced for all women aged 18 and over, regardless of family history of cancer.

3. Please indicate the extent to which you disagree or agree with the following statements.

If population-based genetic testing for ovarian cancer were introduced:

|  | Strongly disagree | Disagree | Neither disagree or agree | Agree | Strongly agree |
| --- | --- | --- | --- | --- | --- |
| It would help identify those with a high risk of ovarian cancer. |  |  |  |  |  |
| My patients could be discriminated against by insurers due to genetic test results. |  |  |  |  |  |
| It could be cost effective in the long-term. |  |  |  |  |  |
| Explaining genetic testing would be too time consuming. |  |  |  |  |  |
| It would help patients to make good healthcare decisions about managing their risk. |  |  |  |  |  |
| It could have a negative impact on some of my patients. |  |  |  |  |  |
| I would be willing to offer all my adult female patients genetic testing for ovarian cancer risk. |  |  |  |  |  |

**OVARIAN CANCER AND GENETICS: KNOWLEDGE**

This section covers what you know about ovarian cancer and genetics. This topic is likely to be very familiar to some groups of clinicians but may be less so for others.

4a. A smear test is not designed to detect ovarian cancer:

True

False

Not sure

4b. Taking the contraceptive pill can increase a woman’s risk of developing ovarian cancer:

True

False

Not sure

4c. Most cases of ovarian cancer are caused by an inherited predisposition:

True

False

Not sure

4d. Paternal family history of cancer is as important as maternal family history of cancer when considering a patients risk of ovarian cancer:

True

False

Not sure

4e. A test result that shows a patient has a genetic Variant of Uncertain Significance (VUS) indicates that the patient does not have an increased risk for ovarian cancer:

True

False

Not sure

4f. The average risk of a women developing ovarian cancer in her lifetime is approximately:

2%

6%

10%

12%

14%

Not sure

4g. The risk of a women with a BRCA1 mutation developing ovarian cancer in her lifetime is approximately:

0-10%

10-30%

30-60%

60-80%

80-100%

Not sure

4h. The risk of a woman with a BRCA2 mutation developing ovarian cancer in her lifetime is approximately:

0-10%

10-30%

30-60%

60-80%

80-100%

Not sure

**OVARIAN CANCER AND GENETICS: STRATIFYING RISK**

Risk stratification is an approach by which women could be divided into groups on the basis of their ovarian cancer risk, and managed accordingly. The average lifetime risk for ovarian cancer is approximately 2%.

We are interested in what you think the boundaries are for ‘low risk’, ‘intermediate risk’ and ‘high risk’ for ovarian cancer.

5a. Please indicate the level of lifetime risk (as a percentage) that you think is ‘**low risk**’:

Below______%

5b. Please indicate the level of lifetime risk (as a percentage) that you think is ‘**intermediate risk**’:

Please enter a range.

____________

5c. Please indicate the level of lifetime risk (as a percentage) that you think is ‘**high risk’**:

Above______%

**CANCER RISK STRATIFICATION**

**Information:

Based on previous research and expert knowledge, the ovarian cancer risk boundaries for PROMISE have been set as:
• Low: 0 to 4.9% lifetime risk.
• Intermediate: 5% to 9.9% lifetime risk.
• High: 10% and greater lifetime risk.**

6. Please indicate the extent to which you disagree or agree with the following statements concerning risk stratification:

|  | Strongly disagree | Disagree | Neither disagree or agree | Agree | Strongly agree |
| --- | --- | --- | --- | --- | --- |
| Risk stratification would help identify those most in need of screening for ovarian cancer. |  |  |  |  |  |
| Risk stratification would result in missing ovarian cancer in some patients. |  |  |  |  |  |
| Risk stratification for ovarian cancer would give patients a sense of control over their health. |  |  |  |  |  |
| Risk stratification for ovarian cancer would make patients feel fatalistic about their health. |  |  |  |  |  |
| Stratification into a group with a **low risk** of ovarian cancer would give patients a false sense of security. |  |  |  |  |  |
| Stratification into a group with a **low risk** of ovarian cancer would be reassuring for patients. |  |  |  |  |  |
| Stratification into a group with **high risk** would have a negative impact on patients’ psychological well-being. |  |  |  |  |  |
| Stratification into a group with **intermediate risk** would have a negative impact on patients’ psychological well-being. |  |  |  |  |  |
| Stratification into a group with **low risk** would have a negative impact on patients’ psychological well-being. |  |  |  |  |  |
| I am confident that I could explain what ‘**low risk’** means to patients in that group. |  |  |  |  |  |
| I am confident that I could explain what ‘**intermediate risk’** means to patients in that group. |  |  |  |  |  |
| I am confident that I could explain what **‘high risk’** means to patients in that group. |  |  |  |  |  |

*Risk stratification involves identifying individuals’ risk of disease from a variety of sources of information and grouping them into defined stratum accordingly.

**INTERVENTIONS**

Information

Women taking part in the PROMISE programme will be offered an intervention tailored to their risk group:
• Low risk: lifestyle advice and information to increase their knowledge about ovarian cancer symptoms.

• Intermediate risk: 4-monthly screening with biomarker (CA125) and annual transvaginal ultrasound screening for women aged >40, or surgery (bilateral salpingo-oophorectomy) if they are aged over 40 years and carry a pathogenic gene mutation.

• High risk: risk reducing surgery as the primary option, or biomarker (CA125) screening every four months and annual transvaginal ultrasound screening if they are aged over 35.

7. We are interested in how willing you would be to discuss the suggested interventions with patients if a programme like PROMISE was made available. Please indicate below:

|  | No, definitely not | No, probably not | Yes, probably | Yes, definitely |
| --- | --- | --- | --- | --- |
| Would you be willing to discuss lifestyle advice and symptom awareness information to patients with a **low risk** of ovarian cancer? |  |  |  |  |
| Would you be willing to discuss the suggested screening intervention to patients with an **intermediate risk** of ovarian cancer? |  |  |  |  |
| Would you be willing to discuss the suggested surgery intervention to patients with an **intermediate risk** of ovarian cancer? |  |  |  |  |
| Would you be willing to discuss the suggested screening intervention to patients with a **high risk** of ovarian cancer? |  |  |  |  |
| Would you be willing to discuss the suggested surgery intervention to patients with a **high risk** of ovarian cancer? |  |  |  |  |

*Bilateral salpingo-oophorectomy is the surgical removal of both the ovaries and the Fallopian tubes.*

**INTERVENTIONS – LOW RISK**

You indicated that you would **definitely not/probably not/probably/definitely** be willing to discuss lifestyle advice and symptom awareness information with patients with a low risk of ovarian cancer.

We are interested in why you made this decision, please provide a brief explanation:

(Max 250 characters)

**INTERVENTIONS – INTERMEDIATE RISK**

You indicated that you would **definitely not/probably not/probably/definitely** be willing to discuss the suggested (CA125 blood test and ultrasound) screening intervention with patients with an **intermediate risk** of ovarian cancer.

We are interested in why you made this decision, please provide a brief explanation:

(Max 250 characters)

You indicated that you would **definitely not/probably not/probably/definitely** be willing to discuss the suggested surgery (bilateral salpingo-oophorectomy) intervention with patients with an **intermediate risk** of ovarian cancer.

We are interested in why you made this decision, please provide a brief explanation:

(Max 250 characters)

**INTERVENTIONS – HIGH RISK**

You indicated that you would **definitely not/probably not/probably/definitely** be willing to discuss the suggested (CA125 blood test and ultrasound) screening intervention with patients with a **high risk** of ovarian cancer.

We are interested in why you made this decision, please provide a brief explanation:

(Max 250 characters)

You indicated that you would **definitely not/probably not/probably/definitely** be willing to discuss the suggested surgery (bilateral salpingo-oophorectomy) intervention with patients with a **high risk** of ovarian cancer.

We are interested in why you made this decision, please provide a brief explanation:

(Max 250 characters)

**BACKGROUND QUESTIONS**

8. What is your gender?

Male

Female

Prefer not to say

9. What is your age? ______

10. What is your ethnicity?

White British

White Irish

Any other White background

White and Black Caribbean

White and Black African

White & Asian

Any other mixed background

Indian

Pakistani

Bangladeshi

Any other Asian background

Black Caribbean

Black African

Any other Black background

Chinese

Other

Prefer not to say

If other, please specify: _______________________

11. What is your profession?

General Practitioner

Geneticist

Gynaecologist

Gynae - oncologist

Nurse specialist

Oncologist

Oncology Surgeon

Other

If other, please specify: _______________________

12. What clinical setting do you work in?

General Practice

Genetics clinic

Hospital

Other

If other, please specify: ________________________

13. How many years have you worked in this profession? __________

14. Which year did you graduate from training to work in your current profession? __________

15. During training did you learn about inherited cancer risk?

Yes

No

Not sure

16. How often are you involved with the referral or any other part of the process of assessing risk for ovarian and/or breast cancer?

Never

Rarely

Sometimes

Often

17. Have you or any of your close relatives been diagnosed with ovarian or breast cancer?

Yes

No

Not sure

Prefer not to say

18. Have you or any of your close relatives ever had genetic testing for cancer risk?

Yes

No

Not sure

Prefer not to say
