# Supplementary material for: Health care professionals’ attitudes towards population-based genetic testing and risk-stratification for ovarian cancer: a cross-sectional survey
Source: BMC Womens Health. 2017 Dec 16;17:132. doi: 10.1186/s12905-017-0488-6 (PMC5732525; doi:10.1186/s12905-017-0488-6)
Supplement: Supplementary file 2 — Comparisons between healthcare specialists’ ovarian cancer and genetics knowledge and self-efficacy to conduct a cancer risk consultation. Table S2. Comparisons between healthcare specialists willingness to offer all adult female patients genetic testing for ovarian cancer risk. Table S3. Willingness to discuss risk stratified interventions with patients. Table S4. Reasons for lack of willingness to discuss suggested interventions. Table S5. Reasons for lack of willingness to discuss suggested interventions (DOCX 31 kb) [file 12905_2017_488_MOESM2_ESM.docx]

**Supplementary Data Tables**

Table S1: Comparisons between healthcare specialists’ ovarian cancer and genetics knowledge and self-efficacy to conduct a cancer risk consultation (n=136).

|  | Genetics specialists (n=44) | Oncologists  (n=45) | Gynaecologists  (n=15) | General Practitioners (n=32) | H | p |
| --- | --- | --- | --- | --- | --- | --- |
| Self-efficacy in conducting a cancer risk consultation total |  |  |  |  |  |  |
| Median (IR) | 3.9 (0.3)^a^ | 3.3 (0.6)^b^ | 3.1 (1.0)^b^ | 2.4 (0.6)^c^ | 84.273 | .000 |
| Ovarian cancer and genetics knowledge total |  |  |  |  |  |  |
| Median (IR) | 8.0 (1.0)^a^ | 7.0 (1.0)^b^ | 7.0 (2.0)^a, b^ | 4.0 (2.0)^c^ | 73.233 | .000 |

IR = Interquartile range.

Corresponding superscript letters denote scores that do not significantly differ from each other at p<.05 level.

Table S2: Comparisons between healthcare specialists willingness to offer all adult female patients genetic testing for ovarian cancer risk (n=136)

|  | Genetics specialists (n=44) | Oncologists  (n=45) | Gynaecologists (n=15) | General Practitioners  (n=32) |
| --- | --- | --- | --- | --- |
| Disagree, n (%) | 28 (63.6)^a^ | 4 (8.9)^b^ | 3 (20.0)^b^ | 4 (12.5)^b^ |
| Neutral, n (%) | 8 (18.2)^a^ | 10 (22.2)^a^ | 5 (33.3)^a^ | 12 (37.5)^a^ |
| Agree, n (%) | 8 (18.2)^a^ | 31 (68.9)^b^ | 7 (46.7)^a, b^ | 16 (50.0)^b^ |

Corresponding superscript letters denote column proportions that do not significantly differ from each other at p<.05 level.

Table S3: Willingness to discuss risk stratified interventions with patients (n=146)

|  | No, definitely not  N (%) | No, probably not  N (%) | Yes, probably  N (%) | Yes, definitely  N (%) |
| --- | --- | --- | --- | --- |
| Willingness to discuss: |  |  |  |  |
| Lifestyle advice & symptom awareness to ‘low risk’ patients. | 5 (3.4) | 12 (8.2) | 51 (34.9) | 78 (53.4) |
| Screening for ‘intermediate risk’ patients | 5 (3.4) | 17 (11.6) | 56 (38.4) | 68 (46.6) |
| Surgery for ‘intermediate risk’ patients | 4 (2.7) | 19 (13.0) | 55 (37.7) | 68 (46.6) |
| Screening for ‘high risk’ patients | 7 (4.8) | 19 (13.0) | 39 (26.7) | 81 (55.5) |
| Surgery for ‘high risk’ patients | 5 (3.4) | 9 (6.2) | 40 (27.4) | 92 (63.0) |

Table S4. Reasons for lack of willingness to discuss suggested interventions

| Themes | Sample quote | Low risk information giving  (n = 15) | Intermediate risk screening  (n = 20) | Intermediate risk surgery  (n=19) | High risk screening  (n=18) | High risk surgery  (n = 7) |
| --- | --- | --- | --- | --- | --- | --- |
|  |  | Frequency | Frequency | Frequency | Frequency | Frequency |
| Practical reasons (lack of time, resources, staff) | “*In my role as a genetic counsellor I would not have support from my managers (or time or resources) to see these patients.”* | 8 | 5 | 4 | - | - |
| Not part of job | “T*hey shouldn't be coming to a genetic clinic to see me at that level of risk. That would be the GP's role”* | 5 | 4 | 6 | 5 | 6 |
| Lack of self-efficacy | “*Not confident I can answer all questions from patient”* | 2 | 2 | 3 | 1 | 1 |
| Risk of patient harm  (physical or psychological) | *“…and may give some women false reassurance after a 'clear' screen that everything is ok.”* | 1 | 1 | 2 | 1 | - |
| Unconvincing evidence/ Unproven | *“There is still insufficient evidence that this screening will detect ovarian cancer at an early enough stage to improve outcome.”* | - | 11 | 3 | 8 | - |
| Inappropriate option | “*The threshold set by our genetics service for offering risk-reducing BSO is approximately 10%, so women at intermediate risk have a lower risk than this”* | - | 1 | 2 | 3 | - |
| Conditional responses | *“If her risk perception is still causing significant anxiety, depending on her age BSO may be deemed appropriate.”* | - | - | 1 | 1 | - |

Frequencies may not add up to ‘n’ as individuals could make more than one comment

Table S5. Reasons for lack of willingness to discuss suggested interventions

| Themes | Sample quote | Low risk information giving  (n = 106) | Intermediate risk screening  (n = 86) | Intermediate risk surgery  (n = 87) | High risk screening  (n = 70) | High risk surgery  (n = 74) |
| --- | --- | --- | --- | --- | --- | --- |
|  |  | Frequency | Frequency | Frequency | Frequency | Frequency |
| Low risk is not no risk | *“It is important that women stratified as low risk understand that this is not the same as 'no risk'…”* | 14 | - | - | - | - |
| Useful information for patients/ opportunity for health promotion | *“It is important to use every opportunity to educate patients…”* | 31 | - | - | - | - |
| Part of job/ already do this or similar | “*I have been discussing this with patients for many years already”* | 19 | 16 | 14 | 11 | 17 |
| Self-efficacy | *“I feel I have enough knowledge to explain this”* | 11 | 11 | 8 | 7 | 4 |
| Patient benefit (physical or psychological) | “*If a woman is classified as at increased risk to general population her level of anxiety may also be greater, having some kind of screening tool to discuss may be helpful”* | 19 | 14 | 15 | 10 | 23 |
| Practical reasons | “*Holistic approach to patient care, every patient counts…”*  *“Not all patients will want to have risk-reducing surgery”* | 3 | 6 | **2** | 3 | - |
| Evidence based/proven intervention | “*Strong evidence for this practice. Simple discussion.”* | 1 | 1 | 7 | 3 | 11 |
| Few or no other options | “*At present, these are the only available modalities and I would be happy to offer patients who are appropriately counselled”* | 1 | 3 | 7 | 2 | 3 |
| Reasonable/  appropriate option | “*Seems a reasonable choice to offer patients”* | 3 | 9 | 2 | 5 | 3 |
| Discuss options/ provide patient (informed) choice | *“To give patient choice and shared decision making”* | - | 9 | 14 | 10 | 8 |
| Conditional | “*I would discuss it as part of a study...”*  “*If screening was of proven benefit I would be happy to be able to discuss this with my patients.”*  “*I would discuss, but probably only for women over 50.”*  “*Happy to do so if given the right information to explain to patients.”* | 12 | 21 | 18 | 15 | 6 |
| Other^^[[1]](#footnote-1)^^ | “*Happy to discuss but as oncologist not likely to be involved with the pre-cancer diagnosis cohort”*  *“Would explain it is not shown to be an effective tool so far”* | 7 other | 3 other  7 discuss pros/cons  8 discuss limitations of screening | 5 other  7 discuss pros/cons | 3 other  1 discuss pros/cons  5 discuss limitations of screening | 8 other  1 discuss pros and cons |
| Miscellaneous^^[[2]](#footnote-2)^^ | *“I don’t know if the interventions we have reduce mortality - no point in screening if you can’t change outcome”* | 7 | 11 | 3 | 5 | 2 |

Frequencies may not add up to ‘n’ as individuals could make more than one comment

1. The theme ‘other’ refers to statements that do not fit under other themes or that do not provide an explanation for participants’ willingness. For example several participants indicated that they would want to discuss pros and cons or the lack of evidence for screening. [↑](#footnote-ref-1)
2. Miscellaneous responses are those that could not be interpreted. [↑](#footnote-ref-2)
